# Effect of Frying Temperature on Lipid Binding, Fatty Acid Composition, and Nutritional Quality of Fish Crackers Prepared from Carp (*Ciprinus carpio* L.) and Tapioca Starch (*Manihot esculentus*)

**DOI:** 10.3390/molecules30051139

**Published:** 2025-03-03

**Authors:** Grzegorz Tokarczyk, Grzegorz Bienkiewicz, Patrycja Biernacka, Sylwia Przybylska, Wojciech Sawicki, Małgorzata Tabaszewska

**Affiliations:** 1 Department of Fish, Plant and Gastronomy Technology, West Pomeranian University of Technology in Szczecin, Papieża Pawła VI 3, 71-459 Szczecin, Poland; grzegorz.tokarczyk@zut.edu.pl (G.T.); sylwia.przybylska@zut.edu.pl (S.P.); 2 Department of Commodity Science, Quality Assessment, Process Engineering and Human Nutrition, West Pomeranian University of Technology in Szczecin, Papieża Pawła VI 3, 71-459 Szczecin, Poland; grzegorz.bienkiewicz@zut.edu.pl; 3 Department of Applied Microbiology and Physiology of Human Nutrition, West Pomeranian University of Technology in Szczecin, Papieża Pawła VI 3, 71-459 Szczecin, Poland; wojciech.sawicki@zut.edu.pl; 4 Department of Plant Products Technology and Nutrition Hygiene, University of Agriculture in Krakow, Balicka 122, 30-149 Krakow, Poland; malgorzata.tabaszewska@urk.edu.pl; 5 Department of Human Nutrition and Metabolomics, Pomeranian Medical University in Szczecin, Broniewskiego Street 24, 71-460 Szczecin, Poland

**Keywords:** fish crackers, *Ciprinus carpio*, tapioca starch, fatty acid, nutritional quality

## Abstract

**Background:** The growing consumption of snack foods such as chips driving demand for healthier, more nutritious alternatives. This study investigated the effect of frying temperature on oil absorption, oil binding capacity, and fatty acid composition of fish-based snacks made from a 1:1 ratio of tapioca starch and carp meat obtained after the separation of the remains of its industrial filleting. **Methods:** The snacks were deep-fried at 160 °C, 170 °C, and 180 °C, and analyzed for expansion, oil absorption, oil binding capacity, fatty acid profiles, and nutritional indices. Oxidation levels and free fatty acids were also measured, ensuring compliance with legal limits. **Results:** Deep-frying at 180 °C resulted in significantly higher snack expansion (95.20%) than the 50% expansion observed at 160 °C and 170 °C. However, snacks deep-fried at 180 °C absorbed the most oil (29.07%) and exhibited the lowest oil binding capacity (8.84%), whereas deep-frying at 160 °C and 170 °C led to oil binding capacities of 15.83% and 18.58%, respectively. Fatty acid profiles also showed temperature-dependent changes, with increased oil absorption reducing omega-3 to omega-6 ratios. Importantly, deep-frying for 45 s at all temperatures did not lead to excessive oxidation or free fatty acid levels beyond regulatory thresholds. Nutritional indices of the deep-fried product were comparable to those of vegetable oils, while before deep-frying, they resembled those of seafood products like shellfish and seaweed. **Conclusions:** While higher frying temperatures improve the texture and expansion of fish-based snacks, they also increase oil absorption and reduce oil binding. Based on these findings, deep-frying at 180 °C was suggested as the optimal condition to balance product texture, oil absorption, and nutritional quality, making the snacks a healthier alternative to conventional deep-fried products.

## 1. Introduction

In the food industry, snacks like potato chips, breadsticks, crisps, and crackers are omnipresent, and their popularity continues to grow. In 2023, the retail value of the European savory snacks market amounted to around €40 billion, representing nearly 3.5% of the European food and beverages market. The consumption of savory snacks across Europe varies from country to country but on average around 4 kg were purchased per year per capita [1]. One notable traditional fish-based snack is crackers, which are known by various names across Asia—for example, they are referred to as wafers or crackers in India and “Keropok” in Malaysia [2].

While popular among all age groups, starch-based snacks typically lack high-quality protein and essential minerals. Incorporating fish in appropriate quantities, characterized by high-quality protein and a favorable amino acid profile, significantly enhances the nutritional value of these snacks. The carp (*Cyprinus carpio* L.) is an economically important farmed fish in central Europe and is a good species for this purpose [3]. Carp, in particular, is a valuable source of protein (approximately 16–18 g per 100 g of raw fillet) [4] and essential fatty acids, including omega-3 (EPA and DHA) and omega-6, which play a crucial role in cardiovascular health [5]. Additionally, it provides important micronutrients such as phosphorus, selenium, and vitamins A and D. However, the anatomical structure of carp, which includes a high number of small bones, makes it challenging to process and less favored in traditional fillet-based products [6]. The study was conducted using carp meat obtained after the separation of the remains of industrial filleting. This approach enhances resource efficiency, reduces waste, and retains high-quality protein, essential fatty acids (omega-3 and omega-6), and key micronutrients, making it a sustainable and nutritionally valuable ingredient for fish-based snacks [7]. It was known that the type of flour or starch used to make crackers influences their quality. Various types of starch/flour have been studied [8] for the production of crackers, such as tapioca, rice, or sago starches. From these studies, there was a consensus that sago and tapioca provided better cracker expansion than other starches. Tapioca is considered a base food for humans in many countries and is the main ingredient, alongside comminuted fish flesh, used to make fish crackers [6,9].

Another important problem is the high content of fat, which is largely absorbed in the expansion process during deep-frying, increasing the calorific value of the product [10]. The fat content of deep-fried snacks is usually high, up to 50% [11]. During deep-frying, oil at high temperatures (160–190 °C) undergoes various physical and chemical transformations, such as hydrogenation, oxidation, and polymerization. These changes influence the nutritional value and safety of the deep-fried products [12,13].

In the case of products such as keropok (fish crackers), oil plays a crucial role during deep-frying, not only by aiding in expansion and enhancing texture and sensory qualities but also by interacting with starch. These interactions result in the formation of starch–lipid and starch–lipid–protein complexes, which influence the structural and nutritional properties of the product. Studies indicate that these complexes, particularly those formed with exogenous lipids introduced during deep-frying, can alter starch’s molecular structure into a more compact V-type arrangement, limiting enzymatic digestion and bioavailability [14]. Zhang and Hamaker [15] elucidated the role of starch–lipid complex formation (in binary systems), and starch–lipid–protein complex formation (in ternary systems), suggesting changes related to structural integrity and reduced digestibility. Oil absorption during deep-frying is different from interactions in gelatinization processes. Deep-frying is a high-temperature process in which the oil penetrates inside the product, removing water from it [16]. Oil absorption is affected by initial water content, product shape, surface porosity, frying method, and temperature [17,18]. Deep-frying remains an efficient, convenient, and economical processing method that produces microbiologically safe food with desirable sensory properties, such as color, aroma, and taste [19]. However, high oil absorption during deep-frying has nutritional implications, increasing the total fat content of the product. While the inclusion of fats in the diet is essential for providing energy and supporting physiological functions, the excessive consumption of fats, particularly as part of a high-fat diet—where dietary fat provides more than 35% of daily caloric intake—has been linked to the development of chronic diseases such as obesity, cardiovascular disease, and certain types of cancer [20,21]. It is important to note that these effects are associated with overall dietary patterns rather than the consumption of individual foods in isolation. Therefore, strategies to control oil absorption during deep-frying could help to improve the nutritional profile of deep-fried products without compromising their sensory quality.

The objective of this study was to investigate the effect of frying temperature on the binding of lipids and the fatty acid profile of fish snacks made from carp (*Cyprinus carpio* L.) and tapioca starch (*Manihot esculenta*). Specifically, this research focused on exploring the mechanisms of oil absorption and changes in the fatty acid profile, including the preservation of essential fatty acids such as omega-3 and omega-6. It also aimed to assess the impact of lipid transformations occurring during deep-frying on the oxidative stability of the product, a factor directly related to food safety. Furthermore, this study sought to provide insights into minimizing undesirable fat content while maintaining the nutritional integrity and sensory qualities of the product. The research developed and evaluated a novel fish-based snack utilizing carp and tapioca starch, addressing consumer demand for healthier snack options while preserving desirable sensory and nutritional properties.

## 2. Results and Discussion

### 2.1. Physical and Chemical Quality Assessment

**Figure 1:**
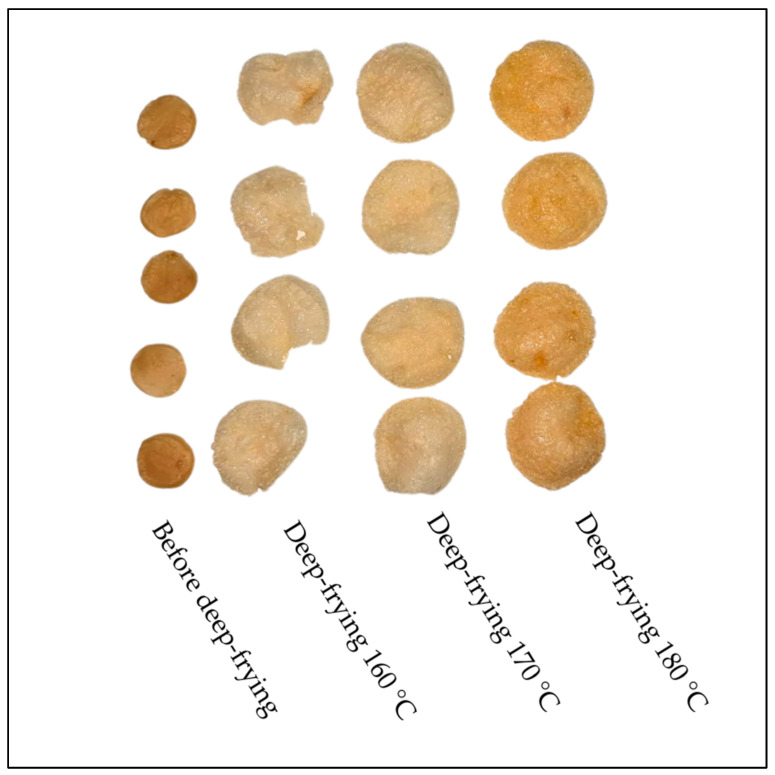
Photographs of crackers prepared from carp and tapioca before and after deep-frying at 160 °C, 170 °C, and 180 °C.

To assess the quality of crackers (Figure 1) prepared from tapioca starch and carp meat obtained after the separation of the remains of its industrial filleting in a 1:1 ratio, the basic physical parameters of deep-fried fish snacks were analyzed in triplicate.

Two key parameters—linear expansion and bulk density (Table 1)—are evaluated, which are directly related to the organoleptic qualities of the snacks, such as their size, texture, and crispiness [9,22]. The results show that deep-frying at higher temperatures, particularly 180 °C, led to a markedly greater linear expansion, nearly doubling the size of the crackers, to lower frying temperatures of 160 °C and 170 °C (54% increase). This expansion is accompanied by a lighter, airier texture. Bulk density is inversely related to frying temperature, with snacks deep-fried at 180 °C exhibiting significantly lower bulk density than those deep-fried at lower temperatures. A reduction in bulk density is typically associated with improved crispiness and mouthfeel, as the product becomes less compact and more porous. This phenomenon can be attributed to the increased evaporation of water at higher temperatures, which accelerates the gelatinization of starch and the formation of a crispy structure, enhancing the textural qualities of the product [23]. The reduction in water content with increasing frying temperature further supports these findings. As the frying temperature increases, the moisture content in the crackers decreases significantly, from 8.58% at lower temperatures to an average of 3.8% at 180 °C. This aligns with previous studies, such as those by Jothi et al. [24], who reported similar trends in vacuum-fried potato chips. The accelerated evaporation of water at higher temperatures is a key factor in achieving the desired texture and expansion in deep-fried products, as it led to the formation of a porous structure with lower moisture content. Additionally, the ash content decreased with the increase in frying temperature, from 2.37 ± 0.07% for crackers deep-fried at 160 °C to 0.91 ± 0.03% for crackers deep-fried at 180 °C. Significant differences in the amount of ash are observed along with increasing the volume and decreasing the bulk density of the snacks [22]. The reduction in ash content observed in the deep-fried crackers is likely due to the migration of mineral components from the meat to the frying oil during the process. This could be explained by the fact that certain minerals, such as calcium and phosphorus (often referred to as lime from bones), may be present in small amounts in the meat used in snack production. Since the meat is recovered from fillet residues, it is possible that minute fragments of bone, which contain these minerals, are present in the product. During deep-frying, the high temperatures could cause these mineral residues to melt and dissolve into the oil [25,26].

### 2.2. Influence of Frying Temperature on Lipid Content and Oil Absorption Dynamics

Table 2 summarizes the results of fat content, and the amount of oil absorbed during deep-frying.

The interaction of lipids with starch and protein is observed already at the stage of cracker production. The fat content in the samples before deep-frying is less than 1.5%, while the fat content in the carp meat was 4.5%. With 50% fish addition, the expected fat content in the cracker before deep-frying is approximately 2.3%, indicating that around 44% of the fat is strongly bound in the starch–lipid–protein system, resisting extraction with a solvent mixture. This binding likely resulted from intensive mixing in the presence of water, facilitating lipid interactions. The formation of inclusion complexes between lipids and starch, particularly with fatty acids like lauric acid, is well documented [27,28,29]. In three-component systems (starch-lipid-protein), interactions become more complex, involving ionic and hydrophobic bonds with proteins [15,28].

Table 2 shows the effect of frying temperature on oil absorption and binding. Oil absorption increased with frying temperature, with statistically significant differences observed between 170 °C and 180 °C. This is closely related to cracker expansion (Table 1). At 160 °C and 170 °C, 15.8% to 18.6% of the oil was strongly bound, but increasing the temperature to 180 °C reduced binding to 8.8%, despite higher oil absorption. This suggests that higher temperatures weaken strong interactions between oil, starch, and protein. The small standard deviations observed in the results reflect the controlled experimental conditions and large sample sizes; however, they may not fully capture the inherent variability of deep-frying processes, which can be influenced by factors such as oil temperature fluctuations, and moisture content. The relationship between oil absorption and fat binding during deep-frying is complex and influenced by several factors, including temperature, microstructure, and time. At higher frying temperatures, such as 180 °C, the rapid evaporation of water from the fish crackers and the increased porosity of the snack’s surface result in higher oil absorption. However, this rapid process also leads to a reduction in the degree of fat binding, as the higher temperature results in a faster expulsion of moisture from the snack, leaving less time for the oil to strongly interact with the starch and protein matrix. In contrast, at lower frying temperatures, the oil has more time to bond with the starch and protein structures, leading to a higher degree of fat binding despite lower overall oil absorption [30].

The microstructure of the crackers also plays a crucial role in these interactions. The innovative crackers with filleted carp meat had a more porous surface compared to traditional keropoks, which likely contributed to increased oil uptake. Studies have shown that oil absorption is a microstructural phenomenon, with porosity and interactions with other components playing key roles [31,32,33,34,35]. However, the rapid structural changes occurring at higher temperatures (such as 180 °C) may cause a decrease in the amount of oil that can be tightly bound to the food matrix. This reduced binding capacity at higher temperatures may explain the lower degree of oil retention at 180 °C compared to lower temperatures, despite the higher overall oil uptake. These interactions between temperature, oil absorption, and fat binding underline the importance of controlling deep-frying parameters to optimize both the sensory and nutritional qualities of the product.

### 2.3. Effect of Frying Temperature on Fatty Acid Content

Similarly to the fat content and oil absorption, the fatty acid composition of fish, oil used for deep-frying, and lipids extracted from fish crackers before and after deep-frying are analyzed at three temperatures. The analysis is performed in triplicate. The total amount of fatty acids in fish is 606.82 mg/g of extracted lipid, while in snacks before deep-frying, it was only 60.76 mg/g, indicating strong interactions between fish lipids and tapioca starch during mixing and gelatinization. After deep-frying, the total amount of extracted fatty acids increases from 779 mg/g at 160 °C to 888 mg/g at 180 °C, reflecting higher oil absorption at elevated temperatures. However, the degree of fatty acid binding decreases with increasing frying temperature (Table 2 and Table 3).

Assuming the total amount of fatty acids in the oil is 100% compared to the total amount of these acids extracted from deep-fried snacks, it is found that the percentage of binding of the total amount of fatty acids depends on the frying temperature (in Table 3) and is similar to that in the case of total fat (Table 2). Comparing the effect of frying temperature on the amount of individual fatty acid fractions, it is found that the least fatty acids were recovered after deep-frying at 160 °C. Comparing the effect of deep-frying temperature on the amount of individual fatty acid fractions, it is found that the least fatty acids were recovered after deep-frying at 160 °C, i.e., saturated fatty acids (SFA) 83%, monounsaturated fatty acids (MUFA) 85%, and polyunsaturated fatty acids (PUFA) 76%, respectively, compared to the oil itself. However, at a temperature of 180 °C, the content of individual groups of fatty acids is, respectively as follows: SFA, 107.5%; MUFA, 91%; PUFA, 98%. The over 100% increase in the SFA fraction was caused by the release of fatty acids from the fish meat, present in the 50% share in fish snacks. These findings align with studies by Wang et al. [36], who demonstrated that water content influences starch–fatty acid complex formation during deep-frying. For samples with 8% water content (similar to the fish snacks in this study), they reported oil complexation degrees of 14.78% for corn starch and 9.21% for rice starch, consistent with the results presented here. Fatty acids could enter the amylose helix, forming more stable amylose–fatty acid complexes [37,38]. However, most of these types of complexes were formed at the stage of mixing the starch solution with lipid and in the process of its gelatinization. These results underscore the importance of optimizing frying conditions to balance sensory attributes with nutritional quality.

### 2.4. Effect of Frying Temperature on Lipid Quality

Deep-frying has a significant impact on the quality of the oil and the quality of the product that absorbs the oil during deep-frying. The degradation of oil may affect fat binding and absorption during deep-frying [33]. The described experiment characterized the oxidation level and degree of hydrolysis of fat lipid extracted from fish snacks prepared from filleted carp meat and tapioca starch (Table 4). The analysis is performed in triplicate. Uniquely, the comminuted fish meat–tapioca composition of the snacks presents a distinctive interaction between fish lipids and starch, which may affect how lipids are absorbed and degraded compared to typical starch-based products such as potato chips. This makes fish-based snacks a novel matrix for studying the lipid degradation process during deep-frying.

The quality of the oil used in this study is maintained at a high level throughout the entire deep-frying process, with a TOTOX index of 18.02 and an acid value of 1.2 mg KOH. These values were notably lower than the permissible limits for oil, as set by the Polish Ministry of Health [39], indicating that the oil remained in a good condition even after repeated use. This suggests that the protein–starch matrix of the fish snacks may protect the oil from excessive degradation, possibly by limiting oxidation. In contrast, oil quality typically deteriorates rapidly in traditional starch-based snacks, often exceeding legal oxidation limits [40]. The result suggests that fish-based snacks may offer a healthier alternative to traditional snacks due to lower degradation of the oil. Increasing the frying temperature above 160 °C reduced the number of primary oxidation products, likely due to their thermal decomposition and complex formation with the starch matrix, consistent with other studies [40,41]. The total oxidation level expressed as TOTOX differed statistically significantly between the oil after deep-frying and the lipid extracted from fish snacks, primarily due to higher levels of secondary oxidation products in the extracted lipids. The acid value (AV) increased during deep-frying, but frying temperature had no significant effect on lipid hydrolysis, likely due to the short frying time. This may indicate that the amylose–protein–lipid complexes in the fish snacks form a hydrolysis-resistant matrix, protecting the oil from breakdown [42,43]. However, a statistically significant difference is observed in the degree of hydrolysis between the oil after deep-frying and the lipids extracted from the product. The lipid extracted from the snacks showed a greater degree of hydrolysis.

### 2.5. The Influence of Frying Temperature on Nutritional Quality Indices of Lipids

Fatty acids, which are often found in dietary fats, can have either beneficial or detrimental effects on disease prevention and therapy. Nutritional indices for fatty acids are very important parameters when assessing the quality of the lipid fraction of food [44]. Table 5 compares the values of these indices for raw fish and finished products before and after deep-frying. The analysis is performed in triplicate. One of the most important indices characterizing the appropriate balance of fatty acids in the diet is the ω-3 to ω-6 PUFA ratio. Dietary recommendations indicate that the optimal ratio in the entire diet is a ratio of 1 to 4. However, ω-6 acids dominate in the Central European diet [45,46]. Therefore, it is important to enrich the diet with products with the highest ω-3 to ω-6 ratio [47]. Such products include fish and dishes prepared from them. Table 5 shows the changes in the value of this index in the deep-frying process. Its threefold decrease is observed compared to raw fish, which is caused by the very high absorption of oil in the final product. However, there was no influence of deep-frying temperature on changes in the value of this index.

Another parameter supplementing this indicator is the amount of long-chain n-3 fatty acids (LC ω-3 PUFA). The DHA/EPA ratio is an important index for evaluating the nutritional quality of fish and fish-based products, as both docosahexaenoic acid (DHA) and eicosapentaenoic acid (EPA) were long-chain omega-3 fatty acids with significant health benefits, particularly in reducing inflammation and supporting cardiovascular health [48]. The ratio of DHA to EPA in the raw fish used in this study is 6.91, indicating a relatively balanced presence of these two essential fatty acids in the comminuted meat carp. Compared to other publications, this is a relatively high result [49,50]. If the differences in the DHA/EPA ratio across the frying temperatures are statistically insignificant, it suggests that frying temperature (within the tested range of 160 °C to 180 °C) does not have a meaningful impact on the relative preservation of DHA and EPA in fish snacks. Further indices characterizing the nutritional value of fat are the index of atherogenicity (IA), which characterizes the atherogenic potential of FAs (fatty acids), and the index of thrombogenicity (IT), which characterizes the thrombogenic potential of FAs, indicating the tendency to form clots in blood vessels. It is shown that the product before deep-frying has parameters similar to those of shellfish [51] or some seaweed [52]. However, the deep-frying process and high oil absorption significantly worsen these indicators. It particularly reduces IT, which takes values typical of rapeseed oils [53]. Both the hypocholesterolemic/hypercholesterolemic (HH) ratio and the health-promoting index (HPI) show significant increases after deep-frying compared to the raw comminuted fish meat. The HH ratio increases from 3.05 to 17.54 (160 °C), 17.37 (170 °C), and 15.27 (180 °C) after deep-frying, while the HPI increases from 2.79 to 12.45 (160 °C), 12.21 (170 °C), and 10.31 (180 °C). These changes are largely due to the absorption of oil, which is rich in monounsaturated fatty acids (MUFA), contributing to the higher values of these nutritional indices. While these data may suggest an improvement in lipid quality, particularly in cholesterol metabolism, this does not necessarily mean that the deep-fried product is healthier than the raw fish. The increase in MUFA, while generally considered beneficial for cardiovascular health [54], replaces some of the more nutritionally critical omega-3 polyunsaturated fatty acids found in fish, such as EPA and DHA. These omega-3 PUFAs are known for their potent anti-inflammatory and cardioprotective effects, and their relative reduction in the deep-fried product means a loss of the specific health benefits associated with consuming fish [55]. Therefore, although the HH and HPI indices are improved, this does not fully capture the degradation in the quality of PUFA, which is essential for the health benefits that fish naturally provide. Furthermore, the process of deep-frying increases the total fat content of the product, resulting in a higher calorific density. Increased oil absorption contributes to the increase in calorific intake, which could have negative metabolic consequences if consumed regularly. Therefore, despite the seemingly improved fatty acid profile suggested by the HH and HPI values, the overall nutritional balance of the deep-fried snack may be less optimal compared to the raw fish. In the case of FLQ, this index is used to evaluate the quality of lipids in terms of their content of long-chain polyunsaturated fatty acids (LC-PUFA), particularly the proportion of omega-3 fatty acids like DHA and EPA in total fat content. For comminuted fish meat, the FLQ is 2.58, indicating the naturally high omega-3 content. After deep-frying, the FLQ decreases. This decrease indicates a significant loss of omega-3 fatty acids due to the deep-frying process. The absorption of oil with no omega-3 reduces the content of these valuable fatty acids in the final product. Studies on deep-fried fish products have similarly shown a loss of omega-3s due to the replacement of fish oil with frying oil, especially when using high temperatures or extended frying times [56].

### 2.6. Effect of Frying Temperature on Sensory Assessment

The results of the sensory evaluation, which is conducted using a 5-point rating scale, show a distinct trend of improved overall sensory quality as the deep-frying temperature rises (Table 6). The sensory analysis is performed by a research panel composed of 10 qualified persons. For instance, snacks deep-fried at 180 °C received the highest scores for color (4.9), taste (4.8), and texture (4.9), reflecting a preference for the crispness and golden-brown color typically associated with higher frying temperatures.

However, the aroma score is significantly lower at 180 °C (4.6 ± 0.1) compared to 170 °C (4.9 ± 0.1), suggesting that higher temperatures might lead to slight aroma degradation, potentially due to excessive oil oxidation or volatile compound loss. This trend aligns with the findings reported in the literature. For example, Durmaz and Yuksel (2021) demonstrated that higher deep-frying temperatures enhanced the color and texture of wheat-based chips due to increased Maillard reaction activity and structural crispness [2]. The moderate decline in aroma at 180 °C, despite improvements in other attributes, might be attributed to the thermal degradation of volatile flavor compounds, as noted in studies on sweet potato crisps and fish frying processes [57].

## 3. Materials and Methods

### 3.1. Materials

Tapioca starch (*Manihot esculentus*) was purchased from Eiamheng Modified Starch Co, Ltd. (EH brand, Thailand). Manageable remains of industrial filleting of farmed carp (*Cyprinus carpio*), such as backbones, collarbones, or parts of fins, were obtained fresh from the fish processing plant in Poland and transported in ice to the laboratory. Fine food salt was purchased from Mariager Salt Specialties A/S (Mariager, Denmark) and sugar was purchased from Krajowa Spółka Cukrowa S.A. (Toruń, Poland).

### 3.2. Sample Preparation

The fish remains were washed with chilled potable tap water and then were passed through the drum separator type NF 13DX having a diameter of holes of 4 mm (Bibun, Japan), and cleaned thoroughly in screw separator type SUM 420 having a diameter of holes of 2.5 mm (Bibun, Japan). That was how clean comminuted fish meat was received. The comminuted fish meat was mixed with starch in a ratio of 1:1, 2% salt, 1% sugar, and 30% water (% by weight based on the total weight of starch and wet fish meat). The ingredients were mixed mechanically (approx. 30 s) using a cutter mixer (Empero, Türkiye) until a smooth paste was obtained. The semi-solid paste was then stuffed into cellulose sausage casings (diameter of 4 cm and 30 cm length) using an electric sausage stuffer 282,083 (Hendi, Poland). The stuffed casings were steamed for 90 min using a convection steam oven iCombi PRO 101E (Rational, Germany). After steaming the steamed pastes were cooled in cold water to minimize shrinkage and chilled for 24 h in a refrigerator at 5 ± 1 °C. The gelatinized semi-product was cut manually into slices about 2 mm thick and dried in Profi Line food dehydrator (Hendi, Poland) at 50 °C for 12 h until moisture content was around 10 ± 2%. The dry slice of fish snack was deep-fried in rapeseed oil (Kruszwica, Poland) at 160 °C, 170 °C, and 180 °C for 10 s using electric fryer Kitchen Line 205,839 (Hendi, Poland). The deep-fried crackers were evaluated for different quality analyses. For each analysis, 3 replicates of 12 chips were prepared for each frying temperature analyzed. Physical determinations included measurements obtained from 36 chips (n = 36). For chemical determinations, 3 parallel replicates were performed from 36 crushed chips (n = 3).

### 3.3. Proximate Analysis

The water content was determined by drying the sample in an oven at 105 °C until a constant weight was achieved. The crude protein content was determined using the Kjeldahl method, and a conversion factor of 6.25 was applied to convert total nitrogen into crude protein. Ash content was determined by ashing the samples in an oven at 550 °C for 8–12 h [58,59,60].

### 3.4. Determination of Linear Expansion (%)

Linear expansion was obtained after deep-frying the dried crackers in rapeseed oil at 160 °C, 170 °C, and 180 °C. The dried cracker was ruled with three lines across using a fine oil marker pen. Each line was measured before and after deep-frying. Measurements were performed on twenty samples from each temperature. The percentage linear expansion was calculated according to the method used by Nurul [61], as follows:

(1) $$Linear\;expansion\left[\%\right]=\frac{Length\;after\;puffing-Length\;before\;puffing}{Length\;before\;puffing}\times 100$$

### 3.5. Determination of Bulk Density (g/cm^3^)

To assess the bulk density of fish snacks at first the volume (v) of the crackers was determined in triplicate in each measurement series using sesame seed displacement according to Sahin and Sumnu [62]. Then, the weight (m) of snacks was determined using an electronic scale RADWAG WTC 2000 (Radwag, Poland). Bulk density (*ρ*) was calculated as follows:

(2) $$\rho \;[g/{cm}^{3}]=\frac{m}{v}$$

### 3.6. Determination of Oil Absorption (%)

The percentage of absorbed oil was calculated according to the method used by Nurul [61] as follows:

(3) $$Oil\;absorption\left[\%\right]=\frac{Weight\;of\;cracker\;after\;deep-frying\;\left[g\right]-weight\;of\;cracker\;before\;deep-frying\;[g]}{Weight\;of\;cracker\;before\;deep-frying\;[g]}\times 100$$

The fish crackers were weighed before and after deep-frying in rapeseed oil, using an electronic scale RADWAG WTC 2000 (Radwag, Poland). Measurements were made on twenty samples from each temperature tested and then the average was taken.

### 3.7. Determination of the Degree of Bounded Oil (%)

The degree of bound oil was calculated by the difference in the content of oil absorbed, determined according to point 3.6, compared to the amount of oil determined via extraction using the Bligh and Dyer method [63], according to point 3.8, as follows:

Bounded oil [%] = Absorbed oil (%) − Bligh and Dyer oil (%)(4)

### 3.8. Determination of Lipid Content

Lipids were extracted using the Bligh and Dyer [63] method. Single-phase lipid solubilization with a chloroform–methanol mixture (1:1) was used. Quantification results were expressed as grams of lipid per 100 g of products.

### 3.9. Determination of Lipids Quality Parameters

The peroxide value (PV) was determined in the lipids extracted using the Bligh and Dyer method [63], according to EN-ISO 3960:2017 [64], based on the iodometric determination of iodine liberated by the peroxides with a starch indicator and a sodium thiosulfate standard solution. Results were expressed as milliequivalents of active oxygen per kilogram of lipids (meqO_2_/kg of lipids).

Anisidine value (AsV) was determined in the lipid extract according to the EN-ISO 6885:2007 [65] method, based on the reaction between α- and β-unsaturated aldehydes and p-anisidine reagent. AsV was expressed as 100 times the absorbance measured at 350 nm (Thermo Scientific, Genesys 20, Waltham, MA, USA) in a 1 cm path length cuvette from a solution containing 10 mg of lipid in 1 mL of reaction medium.

Total oxidation (TOTOX) products were calculated as follows:

TOTOX = 2PV + AsV(5)

The acid value (AV) was determined by the titration of 0.1 N KOH in methanol, according to EN ISO 660:2020 [66]. The results were presented in milligrams of KOH per gram of fat.

### 3.10. Fatty Acids Composition

To obtain the fatty acid methyl esters (FAMEs), the extracted lipids underwent alkaline hydrolysis using 0.5 N sodium methylate (CH_3_ONa) [22]. Subsequently, the FAMEs were separated using a gas chromatography apparatus coupled with a mass spectrometer (Agilent Technologies 7890A, Santa Clara, CA, USA) and equipped with a split/splitless-type injector. The separation conditions for the FAMEs were as follows: a was ysed SPTM 2560 column (100 m length, 0.25 mm inner diameter, and 0.20 μm film thickness, catalog no. 24056); helium was used as the carrier gas with a constant flow rate of 1.2 mL/min; the split ratio was 1:50; the injector and detector temperatures were set at 220 °C; and the programmed furnace temperature was started at 140 °C for 5 min, and then ramped up to 240 °C at a rate of 4 °C/min. The total analysis time was 45 min.

The qualitative interpretation of chromatograms was based on comparing the retention times and mass spectra of the specific FAMEs in the sample with those of analogous FAME standards provided by Sigma Company, Tokyo, Japan (Lipid Standard). C19:0 (Merck, Poland) was used as the internal standard for quantification purposes.

### 3.11. Nutritional Quality Indices of Lipids

Indices were calculated using fatty acid profiles. The n-3/n-6 index was obtained by dividing the omega-3 by the omega-6 fatty acids. The DHA/EPA index indicates the ratio between docosahexaenoic acid (DHA) and eicosapentaenoic acid (EPA). The PUFA/SFA index represents the ratio of polyunsaturated fatty acids (PUFA) to saturated fatty acids (SFA).

The index of thrombogenicity (TI) [67] was calculated as follows:

(6) $$TI=\frac{C14:0+C16:0+C18:0}{\left(0.5\times \sum MUFA\right)+(0.5\times \sum n-6PUFA)+(3\times \sum n\;-\;3PUFA)+(\frac{n-3}{n-6})}$$

This index of atherogenicity (IA) [67] was calculated as follows:

(7) $$IA=\frac{C12:0+\left(4\times C14:0\right)+C16:0}{\sum MUFA}$$

The hypocholesterolemic/hypercholesterolemic ratio (HH) [68] more accurately reflects the effects of fatty acids on cardiovascular health compared to the PUFA/SFA ratio and was calculated as follows:

(8) $$HH=\frac{C18:1n-9+C18:2n-6+C18:3n-3+C20:4n-6+C20:5n-3+C22:5n-3+C22:6n-3}{C14:0+C16:0}$$

The health-promoting index (HPI) [44] was calculated as follows:

(9) $$HPI=\frac{\sum MUFA}{C12:0+4\times C14:0+C16:0}$$

The quality of fish lipids (FLQ) [69] was calculated as follows:

(10) $$FLQ=\frac{100\times (C22:6n-3+C20:5n-3)}{\sum Total\;FA}$$

### 3.12. Sensory Assessment

A panel of ten trained people (n = 10) with no prior history of taste or aroma impairments evaluated good sensory qualities using the hedonic approach. Samples were presented at random and coded with three-digit codes to avoid bias. A 5-point rating system assessed color, taste, aroma, and texture, with 1 denoting “undesirable” and 5 denoting “very desirable”. The results of the profile analysis were shown as polar plots. By PN-ISO 5496:1997 and PN-ISO 3972:2016-07, each panelist underwent evaluation and training in sensory sensitivity [70,71].

### 3.13. Statistical Analysis

All experiments were conducted in three replications. The results were expressed as mean ± standard deviation (SD). Statistical analyses of the results were performed using the Statistica 13.1 program, with a significance level of *p* < 0.05, using the post hoc test of Tukey’s significant difference [72].

## 4. Conclusions

Snack food is gaining an increasingly wider group of supporters, and its sales are still growing. Generally, these types of products are not considered “healthy”, so it is important to enrich these products with easily digestible proteins and high-quality lipids. In addition, it is important to understand the mechanisms of interaction of food ingredients during processing, as they have a significant impact on the calorific value, nutritional value, and sensory characteristics of this type of snack food.

This study showed the influence of the frying temperature of fish cracker products (crackers made of tapioca starch with 50% of farmed carp meat) on physical parameters (degree of expansion) and biochemical parameters (fat content and its interactions), which can serve as a model for similar snacks based on fish and starch. It was found that the frying temperature of 180 °C resulted in almost twice the expansion of the snack than the temperature of 160 °C and its density is much lower, and these parameters have a direct impact on the sensory values of snacks. Moreover, at a higher deep-frying temperature of 180 °C, the product absorbs more oil, but it interacts with starch significantly less than at lower frying temperatures. Higher frying temperature (above 170 °C), for a relatively short time, up to 1 min, does not increase the oxidation level and even have a beneficial effect on reducing the amount of peroxides through their thermal decomposition. The sensory evaluation confirmed that a higher frying temperatures enhanced the overall desirability of the product, particularly in terms of texture and color, which aligns with consumer expectations for deep-fried snacks. The product presented in this work is also characterized by very valuable nutritional indices for assessing fatty acids (IA, IT, HH, HPI, FLQ) for this type of product; however, the deep-frying process causing high oil absorption significantly reduces these indices. Future works suggest the further exploration of methods to reduce oil absorption in deep-fried snacks, while maintaining favorable sensory and nutritional properties, especially through alternative deep-frying techniques or pre-treatments.

## Figures and Tables

**Table 1:** Changes in the content of water, ash, and the value of physical parameters during deep-frying fish crackers.

	Frying Temperature [°C]			
Parameters	160	170	180	Before Deep-Frying
Water [%]	3.86 ± 0.08 ^a^	3.84 ± 0.02 ^a^	3.78 ± 0.03 ^a^	8.58 ± 0.15
Ash [%]	2.37 ± 0.07 ^a^	1.26 ± 0.07 ^b^	0.91 ± 0.03 ^c^	3.84 ± 0.08
Bulk density [g/cm^3^]	0.65 ± 0.12 ^a^	0.26 ± 0.24 ^a^	0.21 ± 0.18 ^b^	1.00 ± 0.04
Linear expansion [%]	54.8 ± 2.5 ^a^	52.8 ± 1.6 ^a^	95.2 ± 2.1 ^b^	

The mean ± SD (standard deviation) within rows with different small letters differs significantly (*p* < 0.05) between frying temperature (n = 3); n—number of samples.

**Table 2:** The influence of frying temperature on changes in the content of fat fractions in the analyzed snacks.

	Frying Temperature [°C]				
Fat Fraction	160	170	180	Before Deep-Frying	Fish
Oil absorption [%] (n = 36)	26.7 ± 2.4 ^a^	24.6 ± 2.1 ^a^	29.1 ± 1.5 ^b^		
Fat content [%] (n = 3)	22.5 ± 0.1 ^a^	20.0 ± 1.3 ^b^	26.5 ± 1.1 ^c^	1.2 ± 0.3	4.5 ± 0.3
Bonded fat [%] (n = 36)	15.8 ± 1.6 ^a^	18.6 ± 1.7 ^a^	8.8 ± 1.3 ^b^		

The mean ± SD (standard deviation) within rows with different small letters differs significantly (*p* < 0.05) between frying temperature; n—number of samples.

**Table 3:** Effect of frying temperature on fatty acid composition (mg fatty acid on 1 g fat) extracted from fish crackers.

			Frying Temperature [°C]			
Fatty Acid	Fish	Before Deep-Frying	160	170	180	Oil
C10:0	0.21 ± 0.04	-	0.11 ± 0.02 a	0.14 ± 0.05 a	0.09 ± 0.03 a	0.10 ± 0.02
C12:0	0.79 ± 0.03	-	0.14 ± 0.06 a	0.12 ± 0.04 a	0.16 ± 0.03 a	0.07 ± 0.01
C14:0	4.27 ± 0.14	-	0.25 ± 0.05 a	0.34 ± 0.11 a	0.33 ± 0.06 a	0.38 ± 0.01
C16:0	80.05 ± 2.24	14.85 ± 0.94	40.02 ± 2.08 a	42.34 ± 2.18 a	51.73 ± 3.01 b	47.8 ± 0.91
C16:1	12.94 ± 1.07	1.57 ± 0.09	1.58 ± 0.11 a	1.73 ± 0.14 a	2.27 ± 0.04 b	1.56 ± 0.03
C17:0	3.61 ± 0.09	-	0.22 ± 0.03 a	0.26 ± 0.04 a	0.31 ± 0.05 a	0.12 ± 0.01
C18:0	238.74 ± 8.33	5.12 ± 0.14	16.22 ± 0.08 a	17.97 ± 0.84 a	21.25 ± 1.09 b	20.22 ± 0.54
C18:1 ω-9	220.04 ± 7.09	27.63 ± 1.09	492.87 ± 9.09 a	510.8 ± 8.88 a	525.89 ± 9.54 a	576.67 ± 4.84
C18:2 ω-6	13.29 ± 0.32	9.16 ± 1.14	153.44 ± 3.07 a	164.85 ± 2.94 b	189.15 ± 4.08 c	195.13 ± 3.15
C18:3 ω-3	1.52 ± 0.11	1.23 ± 0.07	55.05 ± 3.94 a	60.46 ± 4.08 a	73.85 ± 1.07 b	83.8 ± 1.09
C20:1 ω-9	2.61 ± 0.15	1.39 ± 0.07	8.67 ± 0.64 a	9.72 ± 0.64 a	9.63 ± 0.66 a	10.89 ± 0.55
C20:2 ω-6	3.68 ± 0.21	-	5.26 ± 0.12 a	5.86 ± 0.71 a	7.54 ± 1.08 a	7.35 ± 0.14
C20:4 ω-3	4.98 ± 0.43	1.63 ± 0.05	2.4 ± 0.10 a	2.65 ± 0.15 a	3.64 ± 0.35 b	-
C20:5 ω-3	1.98 ± 0.14	-	0.36 ± 0.02 a	0.45 ± 0.08 b	0.46 ± 0.06 b	-
C22:4 ω-6	1.03 ± 0.06	-	0.28 ± 0.01 a	0.26 ± 0.03 a	0.31 ± 0.04 b	-
C24:1 ω-9	1.72 ± 0.08	-	0.15 ± 0.02 a	0.22 ± 0.11 b	0.37 ± 0.14 c	-
C22:5 ω-3	1.67 ± 0.09	-	0.86 ± 0.03 a	0.63 ± 0.04 b	0.39 ± 0.07 c	-
C22:6 ω-3	13.69 ± 0.94	1.18 ± 0.09	1.26 ± 0.09 a	1.30 ± 0.14 a	1.58 ± 0.24 b	-
SFA	327.67 ± 7.06	19.97 ± 0.12	56.96 ± 0.12 a	61.17 ± 0.14 b	73.87 ± 0.24 c	68.69 ± 0.03
MUFA	237.31 ± 3.19	30.59 ± 1.14	503.27 ± 6.94 a	522.47 ± 6.85 a	538.16 ± 7.08 b	589.12 ± 3.05
PUFA	41.84 ± 0.67	13.2 ± 0.08	218.91 ± 0.09 a	236.46 ± 0.11 b	276.92 ± 0.11 c	286.28 ± 0.64
Total FA	606.82 ± 3.38	63.76 ± 0.67	779.14 ± 4.03 a	820.10 ± 3.19 b	888.95 ± 3.54 c	944.09 ± 1.82

The mean ± SD (standard deviation) within rows with different small letters differs significantly (*p* < 0.05) between frying temperature; (n = 3); n—number of samples.

**Table 4:** Effect of frying temperature on fat quality extracted from crackers.

	Frying Temperature [°C]				
Parameters	160	170	180	Before Deep-Frying	Oil
PV [meq O_2_/kg fat]	5.8 ± 0.3 a	3.7 ± 0.2 b	3.4 ± 0.4 b	8.6 ± 1.3	3.1 ± 0.5 b
AsV	1.7 ± 0.1 a	4.2 ± 0.1 b	3.1 ± 0.2 c	4.9 ± 1.4	1.8 ± 0.1 d
TOTOX	13.7 ± 0.6 a	11.8 ± 0.4 b	10.8 ± 1.0 b	22.3 ± 2.3	18.0 ± 1.0 c
AV [mg KOH/g fat]	2.0 ± 0.0 a	2.2 ± 0.3 a	1.9 ± 0.1 a		1.2 ± 0.1 b

PV—peroxide value; AsV—anisidine value; AV—acid value; the mean ± SD (standard deviation) within rows with different small letters differs significantly (*p* < 0.05) between frying temperature; (n = 3); n—number of samples.

**Table 5:** Effect of frying temperature on nutritional indices of fish snacks.

			Frying Temperature [°C]			
Indices	Fish	Before Deep-Frying	160	170	180	Oil
ω-3/ω-6	1.32 ± 0.11	0.44 ± 0.03	0.37 ± 0.03 a	0.38 ± 0.042 a	0.40 ± 0.04 a	0.41 ± 0.11
PUFA/SFA	0.13 ± 0.04	0.66 ± 0.02	3.84 ± 0.22 a	3.87 ± 0.24 a	3.75 ± 0.14 a	4.17 ± 0.09
ω-3 PUFA	23.84 ± 0.84	4.04 ± 0.06	59.57 ± 1.24 a	65.04 ± 1.66 b	79.46 ± 1.19 c	83.80 ± 1.15
Σ LC ω-3 PUFA	22.32 ± 0.46	2.81 ± 0.11	4.88 ± 0.44 a	5.03 ± 0.34 a	6.07 ± 0.33 b	0.00 ± 0.00
ω-6 PUFA	18.00 ± 0.94	9.16 ± 0.08	158.98 ± 3.35 a	170.97 ± 3.76 b	197.00 ± 4.12 c	202.48 ± 6.09
DHA/EPA	6.91 ± 0.06	-	3.50 ± 0.02 a	2.89 ± 0.06 a	3.43 ± 0.04 a	-
IA	0.35 ± 0.03	0.34 ± 0.03	0.06 ± 0.02 a	0.06 ± 0.01 a	0.07 ± 0.01 a	0.06 ± 0.01
TI	1.52 ± 0.09	0.57 ± 0.05	0.10 ± 0.02 a	0.10 ± 0.03 a	0.11 ± 0.03 a	0.09 ± 0.01
HPI	2.79 ± 0.03	2.06 ± 0.02	12.45 ± 0.01 b	12.21 ± 0.03 b	10.31 ± 0.03 a	12.21 ± 0.06
HH	3.05 ± 0.01	2.75 ± 0.03	17.54 ± 0.02 a	17.37 ± 0.02 a	15.27 ± 0.04 b	17.76 ± 0.01
FLQ	2.58 ± 0.02	1.85 ± 0.04	0.21 ± 0.04 a	0.21 ± 0.03 a	0.23 ± 0.02 a	-

ω-3—omega 3; ω-6—omega 6; PUFA—polyunsaturated fatty acids; SFA—saturated fatty acids; Σ LC—sum of long-chain fatty acids; DHA—docosahexaenoic acid; EPA—eicosapentaenoic acid; IA—index of atherogenicity; TI—index of thrombogenicity; HPI—health-promoting index; HH—hypocholesterolemic/hypercholesterolemic ratio; FLQ—quality of fish lipids; the mean ± SD (standard deviation) within rows with different small letters differs significantly (*p* < 0.05) between frying temperature; (n = 3); n—number of samples.

**Table 6:** Effect of frying temperature on sensory evaluation of fish snacks.

	Frying Temperature [°C]		
	160	170	180
Color [1–5 pkt]	4.7 ± 0.1 a	4.7 ± 0.0 a	4.9 ± 0.0 b
Taste [1–5 pkt]	4.2 ± 0.1 a	4.6 ± 0.1 b	4.9 ± 0.1 c
Aroma [1–5 pkt]	3.9 ± 0.0 a	4.9 ± 0.1 c	4.6 ± 0.1 b
Texture [1–5 pkt]	3.8 ± 0.1 a	4.0 ± 0.1 b	4.9 ± 0.0 c

The mean ± SD (standard deviation) within rows with different small letters differs significantly (*p* < 0.05) between frying temperature (n = 10); n—number of samples.

## Data Availability

The original contributions presented in this study are included in the article. Further inquiries can be directed to the corresponding author.
